# Structural Bioinformatics and Deep Learning of Metalloproteins: Recent Advances and Applications

**DOI:** 10.3390/ijms23147684

**Published:** 2022-07-12

**Authors:** Claudia Andreini, Antonio Rosato

**Affiliations:** 1 Consorzio Interuniversitario di Risonanze Magnetiche di Metallo Proteine, Via Luigi Sacconi 6, 50019 Sesto Fiorentino, Italy; andreini@cerm.unifi.it; 2 Magnetic Resonance Center (CERM), Department of Chemistry, University of Florence, Via Luigi Sacconi 6, 50019 Sesto Fiorentino, Italy

**Keywords:** bioinorganic chemistry, metal-binding, structural biology, zinc, iron, copper, transition metals

## Abstract

All living organisms require metal ions for their energy production and metabolic and biosynthetic processes. Within cells, the metal ions involved in the formation of adducts interact with metabolites and macromolecules (proteins and nucleic acids). The proteins that require binding to one or more metal ions in order to be able to carry out their physiological function are called metalloproteins. About one third of all protein structures in the Protein Data Bank involve metalloproteins. Over the past few years there has been tremendous progress in the number of computational tools and techniques making use of 3D structural information to support the investigation of metalloproteins. This trend has been boosted by the successful applications of neural networks and machine/deep learning approaches in molecular and structural biology at large. In this review, we discuss recent advances in the development and availability of resources dealing with metalloproteins from a structure-based perspective. We start by addressing tools for the prediction of metal-binding sites (MBSs) using structural information on apo-proteins. Then, we provide an overview of the methods for and lessons learned from the structural comparison of MBSs in a fold-independent manner. We then move to describing databases of metalloprotein/MBS structures. Finally, we summarizing recent ML/DL applications enhancing the functional interpretation of metalloprotein structures.

## 1. Introduction

Living organisms require a variety of metal ions for their optimal functioning [1,2]. The roles of metal ions in cellular and biochemical processes are many, including the stabilization of the three-dimensional (3D) structure of macromolecules, direct participation in the catalytic mechanism of enzymes, the transfer of electrons to/from other molecules, and the regulation of biological processes. In line with their importance, the concentration of metal ions in the cell is tightly regulated [3,4]. This relies upon the combined action of transport, delivery, storage, detoxification, and efflux machineries. Bacterial pathogens share the same requirements for metal ions as all other organisms [5], which they need to acquire from the host organism. Thus, the host can deploy a protective mechanism, called ‘nutritional immunity’, which inhibits the growth of pathogens by limiting the availability of crucial metal ions. A similar related strategy can be pursued through pharmacological treatment [6].

This review will focus on proteins requiring one or more metal ions to be able to carry out their biological function, or for the achievement of their correct fold. These are known as metalloproteins (MPs). MPs can bind individual metal ions directly into their specific binding sites. In parallel, there is an extensive casuistry of metal-containing cofactors, ranging from polymetallic clusters, which can be homo- (such as iron-sulfur clusters) or hetero-metallic (such as the FeMo cofactor), to organic molecules forming metallic complexes that are then incorporated into the protein (such as cobalamin or protoporphyrin IX). In the PDB, 38% of the entries contain at least one metal ion [7,8], while it has been estimated that no less than 40% of enzymes require metal ions for their biological function. [9,10]. The reactivity and physiological role of metal ions in MPs is largely determined by the local protein structure environment through the modulation of how the metal is positioned in the active site, of how it interacts with the substrate and, for redox-active metals, of its reduction potential [11,12].

Recent years have witnessed a steady growth in the application of bioinformatics methods to the investigation of MPs. In this context, the first area of application has been the prediction of the occurrence of metal-binding sites based only on protein sequence information, as the result of the success of genomics initiatives [13,14,15,16,17]. There are numerous reviews of these methodological developments which have been published recently [18,19,20]. For this reason, sequence-based tools for the prediction of MPs will not be addressed here. A field of application that has received significant attention is the 3D-structure-based prediction of the occurrence of metal sites, which leverages the knowledge about the relative position in space of the amino acids potentially providing donor atoms for metal coordination. Indeed, a significant boost in this kind of method is being received from the success of AlphaFold and AlphaFold2 in the CASP initiatives [21,22,23,24], which have greatly improved the availability of useful 3D structural models for proteins which are not yet characterized experimentally [25,26,27]. With the increase in the number of MP structures available, there is also an increase in the opportunity to apply structural comparison methods to identify functional and/or evolutionary links within and among MP families. The latter is thus another topic of interest for this work. Finally, recent updates regarding databases which are relevant for the study of MPs will be covered, next to recent applications of machine or deep learning methods (ML/DL) to these systems. We focused mostly on the developments achieved in the past decade, except for examples that constituted important conceptual innovations.

## 2. Structure-Based Definition of Metal Binding Sites (MBS)

In this review, we will often refer to the metal binding site (MBS) as a substructure that can be defined in a way such that it is possible to automatically extract MBSs from 3D structures deposited in the Protein Data Bank (PDB) [28]. Different research teams have proposed different definitions, which typically tend to produce comparable results in all downstream analyses of MBSs. Typically, the concept is that of extracting a substructure around the metal ion(s) that represents the macromolecular environment sensed by the metal. This substructure should correspond to the minimal environment determining the function of the metal, i.e., the “minimal functional site” [29].

**Figure 1:**
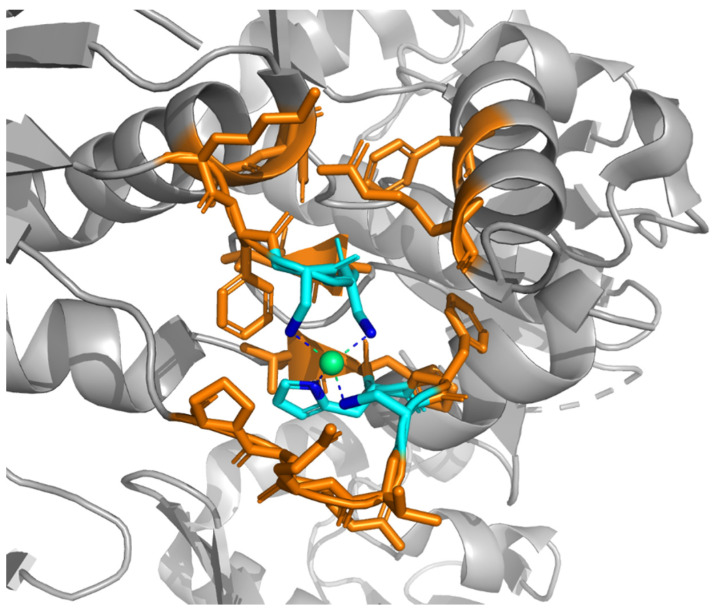
Definition of a MBS according to the MetalPDB protocol. For each metal atom in a given 3D structure, the non-hydrogen non-carbon atoms at a distance smaller than 3.0 Å from the metal ion (green sphere) are identified as its donor atoms (blue atoms), i.e., the atoms that bind directly to the metal. The protein residues or small molecules that contain at least one donor atom are the metal ligands (cyan sticks), and constitute the first coordination sphere of the metal ion. The full MBS is obtained by including any other residue or chemical species with at least one atom within 5.0 Å from a metal ligand (orange sticks). Metal ligands provided by small molecules (e.g., water, ammonia, synthetic inhibitors) or ions (e.g., acetate, hydroxamate) are called *exogenous* ligands. This example MBS is the zinc(II)-binding site of human Schlafen 5 protein (PDB entry 7Q3Z [31]).

In our own work in this field, in order to build an MBS we implemented a protocol that starts with the identification of the metal ion and its donor atoms, i.e., the atoms that form a coordination bond with the metal (Figure 1). Metal ligands are then defined as the amino acids, nucleotides or other chemical entities (e.g., mono- or poly-atomic anions) that contain at least one donor atom (the cyan sticks in Figure 1). The metal ligands provided by protein or nucleic acid residues are called *endogenous* ligands, whereas the metal ligands provided by other chemical entities are called *exogenous* ligands. In proteins, the identity and spacing along the sequence of the amino acid ligands define the metal-binding pattern (MBP) of the metal-binding site. For example, a common MBP in zinc fingers is CX(2)CX(12)HX(2)H, where X denotes any amino acid. In order to extend the selection to include the environment around the metal and its ligands, we add to the MBS any other residue or chemical species with at least one atom within 5.0 Å of a metal ligand (orange residues in Figure 1). A simpler approach to the definition of the MBS adopted by some authors is simply to include any protein/nucleic acid residue or chemical species having at least one atom that is at a distance lower than an arbitrary threshold from the metal (e.g., [30]). In other words, with the latter definition, a sphere of fixed radius is centered on the metal, and the MBS is computed as the ensemble of all of the residues or molecules that have at least one atom contained in the sphere.

Usually, MBSs do not correspond to continuous stretches of a protein sequence. Rather, they are groups of sequence fragments of different lengths, depending on the number and position of the metal ligands, namely the MBP. The fragmented structure of MBSs makes their structural comparison not always possible with standard tools for protein structure superposition, contingent on the specifics of the algorithm used by each tool. Therefore, in some cases, ad hoc approaches have been developed (Section 4).

## 3. Structure-Based Prediction of Metal Sites

### 3.1. Template-Based Methods

**Figure 2:**
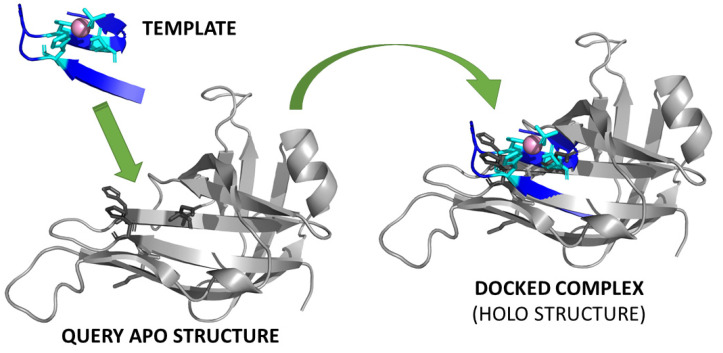
The concept of the template-based detection of MBSs. Each template MBS from a suitably designed library, such as all of the MBSs from the non-redundant PDB, is docked to the query apo-structure. If the docking is successful, the position of the site and the nature of the bound metal are predicted. Often, the docking procedure is guided by first identifying candidate metal ligands in the query structure. The template MBS is the manganese(II)-binding site of the LigD phosphoesterase domain of *Pseudomonas aeruginosa* (PDB entry 3N9B [34]); the apo-structure is taken from PDB entry 2LJ6, which is the solution structure of the same protein in the absence of manganese(II) [35].

The first proofs of concept of the feasibility of using 3D structures of apo-proteins (i.e., not containing the metal ion) to identify MBSs date to the 1990s. They focused on the analysis of a function measuring the contrast between the hydrophobicity of the metal site itself and the surrounding protein residues (a contrast function) [32]. In practice, the contrast function measures the extent to which the outer atoms in a sphere are more hydrophobic than the inner atoms; higher values are associated with spheres centered at MBSs. Thus, the predictions were based on the identification of cavities defined by templates of triads of amino acids with suitable relative spatial arrangements and featuring high hydrophobicity contrast [33]. The idea of matching an apo-structure against a set of templates (Figure 2), i.e., pre-arranged spatial distributions of potential metal ligands, is a very logical approach to leverage both the availability of a structural model for the protein of interest and the information stored in the PDB on existing MPs.

In this direction, a very successful implementation was that of the CHED algorithm [36]. CHED focuses on four residue types—namely Cys (C), His (H), Glu (E) and Asp (D) (hence the acronym)—and searches the query apo-structure for suitable arrangement(s) of triads of the CHED residues. In these arrangements, the donor atoms are at distances from each other which are consistent with the cut-off values taken from the analysis of the MBSs in the PDB. Some possible structural rearrangements are also taken into account by looking for alternative conformations of one side chain at a time. The initial hits are then re-examined using two different filters to remove false positive predictions. A relatively similar approach taking into account the volume available to the side chains of the candidate metal ligands was proposed by Goyal and Mande [37].

IonCom [38] has integrated four existing structure-template-based predictors for general ligand binding, also exploiting the local similarity to templates, with a novel sequence-based predictor, called IonSeq, to predict the binding sites of metal ions as well as of other ions from 3D structural models generated by the i-Tasser modelling tool [39]. Among the template-based predictors used, COACH [40] afforded a particularly relevant contribution. The latter is a consensus method, combining an approach leveraging the 3D alignment of binding-site substructures (TM-SITE), an approach based on sequence profile alignment (S-SITE) and the output of other structure-based predictors using a SVM method. The combined approach achieved an MCC that was 12.5% higher than the individual predictors. A major advantage of IonCom over COACH was due to the integration of complementary sequence-based and template-based methods. The authors noted that the accuracy of IonCom is lower for some metal ions with highly variable MBSs, e.g., alkali metals. This may actually be a general issue, as many tools indeed focus only or mainly on transition metal ions.

The MIB server for the prediction of MBSs and the docking of metal ions [41] uses the fragment transformation method [42] to predict the location of MBS for twelve different metals, and docks the appropriate metal ion (one among Ca^2+^, Cu^2+^, Fe^3+^, Mg^2+^, Mn^2+^, Zn^2+^, Cd^2+^, Fe^2+^, Ni^2+^, Hg^2+^, Co^2+^, and Cu^+^) into the query 3D structure. In order to generate the library of templates, the structures of MPs containing at least one relevant ion were collected from the PDB and filtered at the 30% sequence identity level. The MBS templates were then extracted by selecting all of the residues with at least one heavy atom within 3.5 Å of the metal ion. Then, by applying the fragment transformation algorithm, clusters of residues in the query apo-structure were matched to the templates of the library. Each identified cluster was scored according to its sequence and structural similarity to the template. In practice, this procedure identifies the best superposition (taking into account sequence similarity) of each template to the query structure, and then ranks the results. Because each template contains also the metal ion, the predicted position of the latter within the structure is readily available.

ZINCCLUSTER focuses on the prediction of zinc-binding sites [43], based on the occurrence within the query protein of the structural patterns detected from the analysis of MBSs present in MPs of a known 3D structure.

The implementation of a predictive algorithm in the GaudiMM modeling suite [44] did not explicitly make use of templates. Instead, the authors implemented a method in which groups of protein residues which are potentially able to coordinate a metal ion are discovered in the query structure by seeking suitable donor atoms within a user-defined distance (3.5 Å in the paper) of the metal ion [45]. When the input is an apo-protein, potential MBSs are initially found by probing the structure for accessible pockets whose center of mass is within 3.5 Å from the β-carbon atoms of three or more amino acids of the CHED group (as in the CHED method described above). The identification of donors is then performed from the pocket center. After defining the candidate metal position and the surrounding donor atoms, a series of geometrical aspects are calculated in order to validate the site prediction. Note that, in practice, this procedure requires that a minimum of three donor atoms are present in the site.

BioMetAll expands upon the concept incorporated as part of the GaudiMM suite (see the preceding paragraph) by making the assumption that the geometric patterns of the protein backbone permit the identification of preorganized MBSs [46]. The structural analysis of the conformation of the backbone, instead of the side chains, should make the predictions less dependent on the high quality of the structure and also on the metal-induced reorganization of the site, which often does not greatly affect the protein backbone [47]. The BioMetAll algorithm starts by placing the apo protein structure in a grid of virtual metal probes. This is achieved by retaining only the coordinates of the Cα, Cβ, C’ and backbone O atoms. These atoms are embedded in a spherical grid of equidistributed probes, with an extra thickness of 8 Å to account for the atoms of the residues at the protein surface. Probes at less than 1.0 Å from any protein atom are removed. For every remaining probe, BioMetAll evaluates which protein residues surrounding it meet the geometric parameters defined by the authors through a statistical analysis of the sites in the MetalPDB database [8]. Restrictions on the minimum number and type of the metal ligands are applied in order to produce a list of valid probes along with their potential ligands. With this procedure, each MBS can be associated to a number of metal probes; furthermore, any protein will be associated with several predicted MBSs. The authors observed that there was a good correlation between the number of probes associated with a predicted MBS and the likelihood that the prediction was correct: in 75% of the cases the most populated solution overlapped with the experimental site.

The flexibility of MBSs has been taken explicitly into account for the predictive method described in [48]. In this work, coarse-grained molecular mechanics was applied to produce meaningful ensembles of 11 structural conformations for each query protein. The ensemble represents the conformational space available to the protein, based on its input structure and the force field used, thus contributing to overcoming the problem of metal-induced rearrangements at the site. Then, the recognition of MBS templates from a predefined library is carried out using geometric hashing. Geometry hashing was chosen because it speeds up the comparison, allowing the software to deal with more structures for a single query. On the other hand, it provides limitations to the minimum number of residues in the MBS, which, in the current implementation, should be at least four. The method includes in the evaluation of the template matches only donor atoms from the amino acid sidechains, in order to simplify the identification of candidate sites in the query structure.

### 3.2. Random Forest Methods

Two recent predictors exploited a random forest algorithm. In this type of approach, a computational model is trained using a set of features (i.e., specific properties) extracted from a large number of positive and negative examples of MBSs. The optimized model is then used without further modifications to classify novel structures. The Zincbindpredict tool employs a random forest classifier that was trained to predict entire zinc-binding sites, as opposed to individual zinc-binding residues [49]. In practice, this tool leverages a portfolio of predictive models, each optimized to detect a specific type of zinc-binding site, where the different types—called ‘families’ of sites in the article—correspond to different metal-binding patterns, e.g., C2H2, H3, etc. The features of each family of sites included sequence-derived properties (inter-residue distance, average hydrophobicity and average number of charges around each residue, both computed over three different windows) and structure-derived properties (various combinations of Cα-Cα as well as Cβ-Cβ distances within the MBS, plus the hydrophobic contrast function) [49]. In order to collect a dataset of negative samples, an arrangement of residues matching the pattern of the family in question was taken from a randomly chosen non-zinc-binding PDB structure, and the corresponding feature vector was created. Only residue combinations where the Cα-Cα distances were all below 30 Å were taken into account, in order to physically exclude sites from the negative dataset. The query structure is thus processed in order to identify the combinations of residues matching the different families of sites included in Zincbindpredict; for each potential site, the feature vector generated from the query sequence and structure is fed to the classifier of the corresponding family in order to evaluate the likelihood that it is a true site.

Although it is not aimed at the identification of MBSs in apo-protein structures, another tool employing a random forest classifier was developed to analyze backbone protein structures to identify suitable positions to introduce metal-binding residues in order to engineer MBSs in proteins (i.e., to artificially design an MP given a protein scaffold of known 3D structure) [50]. In practice, the training set contains features that are based only on the coordinates of the backbone atoms, whereas all of the side chain atoms are removed. The position of the Cβ is recalculated from the backbone coordinates to permit the computation of geometric parameters that are fed into the random forest classifiers. Neglecting the position of the side chain atoms makes it possible to predict the occurrence of potential MBSs independently of the identity of amino acids in the current structure, thereby opening the way towards creating engineered binding sites by introducing the appropriate side chains in the positions highlighted by the tool [50].

## 4. Structural Comparison of the Metal Sites

**Figure 3:**
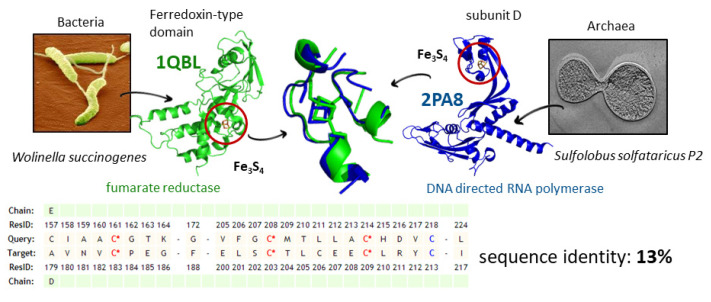
Detection of relationships between MPs with divergent sequences. MBS-driven structural superposition permits the detection of similarities between proteins with no homology, or which have experienced extensive sequence divergence. PDB entry 2PA8 is the crystal structure of the D/L subcomplex of the RNA polymerase of *Sulfolobus solfataricus* [51]. PDB entry 1QLB is the crystal structure of fumarate reductase from the respiratory complex II-like of *Wolinella succinogenes* [52]. This example superposition was computed with the MetalS^2^ [53] tool.

The macromolecular context surrounding the MBS is a major determinant of its chemico-physical properties and, consequently, of its reactivity. The structural comparison of MBSs, therefore, is a tool enabling the functional analysis of MP families and evolutionarily unrelated proteins that harbor similar MBSs. The structural comparison of MBSs is typically performed regardless of the similarity of the global protein fold, in order to focus only on the evolution of the MBSs (Figure 3).

The MetalS^2^ tool (http://metalweb.cerm.unifi.it/tools/metals2/ (accessed on 5 July 2022)) takes as input the structures of two MBSs (as defined in the MetalPDB database) and superimposes them regardless of the protein fold [53]. The very first step of MetalS^2^ is to identify the geometric center of the metal ions (in order to be able to handle polymetallic sites) in each site and then overlap them. Each MBS is then decomposed into triangular units defined by the geometric center of the metals and two donor atoms, and proceeds to systematically superimpose all possible unit pairs from the two sites, always keeping the vertices corresponding to the metal positions coincident. All of the poses generated are ranked according to the MetalS^2^ scoring function, which takes into account both the sequence and structure similarity for the set of relationships defined by each pose. The best ranking poses are optimized by minimizing the RMSD of the Cα and Cβ pairs of the two MBSs, and are re-ranked to provide the final best-scoring superposition [53,54,55]. We subsequently implemented an optimized version of MetalS^2^ to allow users to search the entire MetalPDB database for MBSs that are structurally similar to the site of an MP structure of interest, either taken from the PDB or input by the user. The latter tool, called MetalS^3^, is available as a web server at http://metalweb.cerm.unifi.it/tools/metals3/ (accessed on 5 July 2022) [56]. As an example, in a recent application we used MetalS^3^ to investigate systematically the similarities between the zinc(II)-proteome of SARS-CoV-2 and known metalloprotein structures, uncovering the fact that the large majority of viral MBSs are close to eukaryotic zinc(II) sites [57].

mFASD is a structure-based algorithm that predicts which metal is most likely to populate an MBS [58]. In this tool, the MBS is a represented as a group of functional atoms (functional atoms set, FAS). The local chemical environment of each atom in the FAS is described by integrating information on its chemical properties and the chemical properties of its neighboring atoms. This allowed the authors to define a similarity measure between pairs of FAS atoms. In turn, this enabled the pairwise comparison of FASs by all-versus-all comparison between the atoms in the two sets. A predefined threshold was introduced by analyzing the ROC curve computed for the case of identifying zinc(II) sites in an ensemble of all of the MPs binding one metal among Cu, Fe, Mg, Mn, Zn or Ca, derived from PDBSelect25. mFASD uses this threshold to assign pairs of FASs to the same metal-binding type. Finally, the authors created a reference dataset of FASs, i.e., MBS templates, against which a query MBS could be scanned in order to assign its metal-binding type.

The MeCOM tool also aims at the superimposition of metal sites [59]. The algorithm starts by identifying the metal ion and its protein ligands in each MBS. Then, a metal-centered 40 Å grid is used to create a set of solvent-accessible lattice points, within which the active sites of the MPs are identified. Multiple metal ions within 5 Å of each other are treated as a single cluster when identifying the active site. MeCOM uses the atoms at the surface of the active site to assign specific features to the sites; these features include pharmacophore properties (e.g., the presence of hydrogen bond donor/acceptors) as well as metal coordination and cofactor properties. A quaternion approach is finally deployed to superimpose the two MBSs based on the comparison of their features and the position of the Cα atoms. MeCOM automatically detects and builds MP active sites; this was tested on a dataset of 4223 structures, with a resulting accuracy of 95.5%. Furthermore, a PyMOL plug-in was made available to view and analyze the MeCOM comparison results.

In a recent publication, the already-available TopMatch tool for structure superposition [60,61] was used in conjunction with a novel scoring function called ‘sahle’ [62]. In this work, MBSs are extracted from the MP structures as spheres with a 15 Å radius centered on the metal. The aim of the sahle function, which depends on the length and sequence similarity of the aligned MBSs but not on the RMSD, is to identify functional relationships between the structurally aligned (with TopMatch) MBSs. As TopMatch does not explicitly take into account the metal ions to build the superposition, the authors optimized the parameters in the sahle function to make it capable of detecting structural alignments with a short metal–metal distances between the two superposed MBSs. With the optimized sahle function, the authors could detect the structural similarity of the MBSs in evolutionarily distant MP families, and identified six clusters of ancient metal-binding motifs [62]. Previously, a similar concept was implemented using Pymol as the structure alignment tool and a scoring function based solely on structural similarity parameters [63]. In the latter study, the aims were to define clusters of structurally similar MBSs, also extracted using a 15-Å sphere, and to subsequently link them if members of different clusters co-occurred in the same structure at a distance compatible with an electron-transfer interaction. In this way, a spatial adjacency network (SPAN) was built, based on the structural proximity of MBSs in the electron transfer (ET) chains. The network provided evidence for the existence of four ancient folds that recurred frequently in ET chains.

## 5. Metalloprotein Databases

Numerous databases exist addressing MPs in general or some specific aspects of their chemistry and biology [18]. As with many other databases in biology [64,65,66], a recurring issue is the obsolescence of their contents, even when the sites are still reachable. In this section, we describe some recently created or updated resources on MPs, for which the contents are still current and accessible as of 15 June 2022.

MetalPDB [7,8] is available at https://metalpdb.cerm.unifi.it. MetalPDB (accessed on 5 July 2022). It collects structural information on all of the MBSs present in the PDB and links them to other biological resources such as protein domain databases. For the construction of the MetalPDB database, the MBS is defined as the ensemble of the atoms in the metal ligands and any other atom belonging to a chemical species within 5 Å of a ligand (Figure 1). The website provides extensive statistical analyses on the database’s contents, to facilitate a deeper comprehension of the diversity of the biochemistry of metals. MBSs are grouped into sets of equivalent and equistructural sites, which correspond to sites at a corresponding position within a given protein fold that are populated, respectively, by the same or different metal ions. These groups are linked to apo-protein structures with the same fold, which potentially are missing the metal cofactor.

BioLiP (https://zhanggroup.org/BioLiP/ (accessed on 5 July 2022)) is a semi-manually curated database of molecular adducts involving proteins [67]. The structure contents in BioLIP are harvested from the PDB and cross-referenced with the literature and databases on biological function. Adducts between proteins and metal ions, i.e., MPs, are collected in a specific section of BioLiP.

ZincBind (https://zincbind.net/ (accessed on 5 July 2022)) is a database specializing in zinc-binding sites [68], where the sites were built taking into account the biological assemblies rather than the asymmetric units deposited in the PDB. This is quite relevant for all of the MBSs at the interface between copies of a chain, where taking into consideration only the contribution of a single chain from the asymmetric unit is not biologically correct. ZincBind automatically discards zinc sites that are detected because of crystallization conditions, identified as zinc ions that have less than two protein ligands with three donor atoms. Furthermore, a 90% sequence identity filter was applied to remove redundancy, except when the sites differ in the number, order, or type of protein ligands. The software used to generate the database contents is open source.

Another specialized database is PyDISH (https://pydish.bio.info.hiroshima-cu.ac.jp/ (accessed on 5 July 2022)), which focuses on the analysis of heme-binding sites in PDB structures [69]. PyDISH focuses on the coordination of the heme iron (axial ligands), on the occurrence of the different heme types and on the distortions of the heme porphyrin. Statistical analyses can be obtained from the website. Normal-coordinate structural decomposition [70] was applied to define the porphyrin distortion as displacements from its equilibrium planar structure with D_4h_ symmetry.

VirusMED (https://virusmed.biocloud.top/ (accessed on 5 July 2022)) is a database of epitopes, drug-binding site and metal-binding sites in viral proteins of known 3D structures [71]. For metal-binding sites, this database provides information on the coordination bonds between the protein and the metal ion(s). The enzymatic classification number (EC number) of each polypeptide chain coordinating the metal ion is included in the annotation, along with the taxonomic classification of the virus. A unique feature of VirusMED is that the quality of each site is evaluated using state-of-the-art methods for the validation of metal sites in crystallographic structures [72,73].

InterMetalDB (https://intermetaldb.biotech.uni.wroc.pl/ (accessed on 5 July 2022)) has a focus on MBSs occurring at intermolecular interfaces [30]. As mentioned for ZincBind, a stringent prerequisite to investigate this class of sites is to reconstruct the biological assembly from the asymmetric unit [74]. For the construction of InterMetalDB, intermolecular MBSs were identified by detecting metal ions with donor atoms (within a 3 Å threshold) from at least two protein or nucleotide residues belonging to different macromolecular chains. This criterion explicitly excludes non-macromolecular ligands from the definition. The redundancy of the database contents was reduced by using both a protein sequence filter (at the 50% level) and a clustering approach to identify unique MBSs in structures harboring multiple sites. The analysis of InterMetalDB permitted the identification of metal preferences in interfacial sites as well as the corresponding macromolecular environments [30].

MetLigDB (http://silver.sejong.ac.kr/MetLigDB/home.html (accessed on 5 July 2022)) focuses on the structural and chemical properties of small molecules that bind directly to the metal ion(s) present in MPs [75]. The MetLigDB entries were derived from the analysis of ligand-containing PDB structures. In addition to the structural view of each metal site containing an organic ligand, derived from the relevant PDB entry, the web pages of this database provide information on the binding affinity of the inhibitor for the target MP. This resource is mainly intended to support researchers in the development of novel metal-targeted inhibitors by looking at previously released molecules.

A related, more-recent database is MeLAD (https://melad.ddtmlab.org/ (accessed on 5 July 2022)), which was also derived by extracting from the PDB database all of the 3D structures of metalloenzyme–ligand adducts [76]. MeLAD extends the overview introduced by MetLigDB by integrating the structural view with detailed analyses of the properties of these systems, including metal-binding pharmacophores, metalloenzyme structural similarity and ligand chemical similarity. For example, MeLAD divides organic metal ligands into monodentate, bidentate and tridentate chemotypes, which are then linked to different metal ions and coordination modes. The analysis of the chemical similarity between ligands allowed MeLAD to identify groups of *exogenous* ligands harboring common metal-binding moieties. In turn, these associations are leveraged to cluster the metalloenzymes whosr metal sites interact with the ligands of a given group. Besides their relevance to the development of novel metal-targeted inhibitors, the contents and underlying ideas of MeLAD provide hints to understand *exogenous* ligand selectivity in the context of the entire metalloproteome.

A missing database in the field of metal-based medicinal chemistry is one on metallodrugs (e.g., metallodrug-DB) [77]. Metal-containing complexes formed by small organic molecules are used as effective pharmaceuticals in a variety of contexts, from cancer treatment to antimicrobial and diagnostic agents. There are a number of subtleties associated with metallodrugs, starting with the cytotoxicity of free metal ions, which require a proper understanding of the molecular basis of their action mechanisms [78]. A metallodrug database could address not only metallodrugs which are already approved for clinical use or under clinical development but also harvest information on metal-based compounds tested in relevant biochemical and cellular assays.

## 6. AI Methods Applied to Metalloproteins

ML and DL methods have gained great popularity in the investigation of the 3D structure and reactivity of proteins, and the field of bioinformatics studies of MPs is no exception [79]. In particular, the application of DL to MP structures is relatively recent, in spite of the extensive information available on these systems and on their biological relevance. In line with the rest of this contribution, we do not address methods for sequence-based detection of metal-binding sites here.

A pioneering application of DL to MPs is the use of conditional variational autoencoders for the insertion of metal-binding sites in non-metal-binding proteins without human input [80]. The developed methodology was able to design protein sequences that matched specified attributes, such as metal-binding. The performance of this method was evaluated in comparison to a predictor based on hidden Markov models (HMMs), by estimating the stability of the predicted novel metal-binding structures. This analysis showed that the former approach could generate substantially more stable structures.

At the functional level, a relevant application is the investigation of disease-related mutations through a multichannel convolutional neural network (MCCNN) [81]. The MCCNN was trained using spatial and sequential features for each selected MBS (including both positive and negative examples, i.e., sites with and without known disease-associated missense mutations). The positive examples in the training set included 1256 disease-associated mutations related to ten metals, identified by integrating the information contained in clinical and human genetics databases with the MBSs of MetalPDB. The selected features input to the network included the occurrence of aliphatic and aromatic carbon atoms, the presence of hydrogen bond donors and acceptors, computed interaction energies with the MBS, the physicochemical properties of the aminoacids in the MBS, and data on the mutation. The trained MCCNN can predict disease-associated mutations in both the first and second sphere of MBSs [74] with a very satisfactory performance.

**Figure 4:**
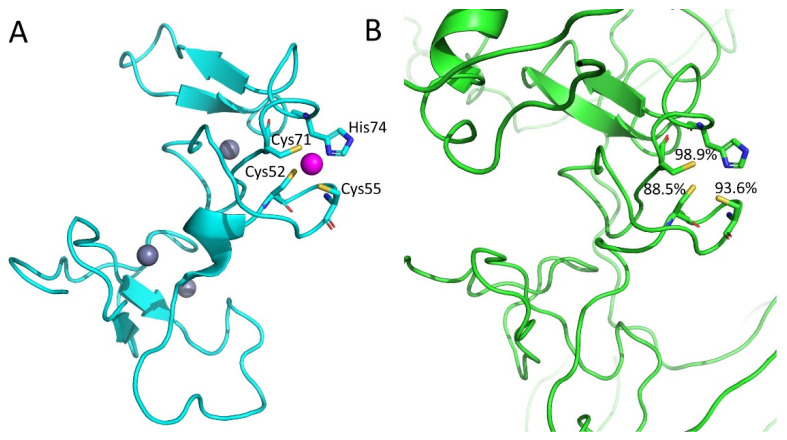
Comparison of a mouse zinc(II)-binding domain with the AlphaFold prediction of the whole structure and analysis of the site with DeepCys. (**A**) The first model of the NMR structure of the PHDVC5HCHNSD1 tandem domain of the Nsd1 protein from *Mus musculus*, with one of its four zinc(II) MBSs highlighted (the metal ion is in magenta) (PDB entry 2NAA [83]). (**B**) AlphaFold model of the entire human homolog of this protein (Uniprot entry Q96L73), with the corresponding site highlighted. The 2NAA structure was input to DeepCys to predict the likelihood that each of the three cysteines is metal-binding; this is mapped onto the model. Some parts of the model were omitted for clarity.

The DeepCys tool uses a NN to predict the probabilities of four different cysteine roles, i.e., metal-binding, disulphide formation, sulphenylation and thioether [82]. The most probable function of each cysteine in the input structure can then be assessed (Figure 4). In particular, the network learned how to identify metal-binding cysteines thanks to the inclusion in the training dataset of PDB structures binding Zn^2+^, Cu^2+^, Cd^2+^, Fe^2+^/Fe^3+^ or Hg^2^ ions. The input features to train the NN included descriptors of the cysteine microenvironment, the secondary structure, the protein family, the hydrophilicity around each cysteine as defined by the protein residues in contact with it, and the occurrence of specific patterns (e.g., CC, CSC, CXXC, etc.). The accuracy of DeepCys for its four different predictions ranged between 75% (thioether) and 87% (disuplhide). Structure-based predictions by DeepCys are only applicable to structures deposited in the PDB.

MAHOMES is a recently developed approach aimed at distinguishing between enzymatic and non-enzymatic metals in MPs [84]. In this work, the authors applied fourteen different machine learning methods, including a neural network approach. These fourteen algorithms were trained on the same data, and the Matthews correlation coefficient (MCC) was the selection criterion to identify the best-performing approach. The MCC is a performance measure that is not particularly sensitive to imbalances in the training set, as non-enzymatic data were about three times the enzymatic data in the input dataset. The best-performing method was an extra trees algorithm, with which MAHOMES achieved a 94.2% accuracy on a validation dataset composed by enzyme structures deposited in 2018 or later. The input features used in MAHOMES included Rosetta energy terms, information on the MBS geometry, a description of the residues defining the MBS, electrostatic energy terms, and coordination geometry information, for a total of 391 features. Information on sequence conservation or the secondary structure was not included by design in order to avoid potential biases towards specific folds. A further interesting outcome of this work was the analysis of which features were more important to discriminate the two categories of sites. The Rosetta energy summed over the spherical volume of the MBS was the most distinctive feature. The other most important features were based on the number and volume of side chain and backbone atoms lining the MBS, showing that enzymatic MBSs had larger volumes and involved a larger number of residues.

An “indirect” use of artificial intelligence in the study of MPs is the exploitation of AlphaFold [21,85] or RoseTTAFold [22] predictions to model or predict the occurrence of MBSs. In fact, the structural models in the AlphaFold database do not contain chemical entities other than natural aminoacids, even when the presence of the cofactor would be required to achieve the proper folding of the polypeptide chain, and also do not take into account the quaternary structure [86,87]. The latter issue was already addressed by RoseTTAFold, and is also being tackled by a novel version of AlphaFold called ‘AlphaFoldMultimer’ [88]. The AlphaFill database [89] aims at filling the gap regarding cofactor-binding to the models in the AlphaFold database by docking small molecules and ions that were observed in complexes with homologous proteins in experimental structures from the PDB-REDO [90,91] repository. In practice, the AlphaFill algorithm uses BLAST [92] to identify close homologs of each AlphaFold model among the PDB-REDO structures that contain a metal ion (or another common cofactor, excluding crystallization agents or metals typically used in heavy-metal derivatives to help phasing). The residues surrounding the cofactor (i.e., the MBSs) in the BLAST hits are used for a local structural alignment of each PDB-REDO structure to the AlphaFold model, thereby allowing the placing of the cofactor within the latter model. The resulting holo-structures are available from the AlphaFill interface (https://alphafill.eu/ (accessed on 5 July 2022)).

In a related work, a thorough search of zinc and iron-sulfur-binding sites was performed on all of the AlphaFold models [93]. The results hinted at the occurrence of a large variety of novel sites that could be predicted thanks to the availability of the 3D models (Figure 4B). The protocol starts with the identification of the coordinates of all of the sidechain or backbone atoms (e.g., the Sγ of cysteine or the Nδ1 and Nε2 of histidine). These potential donor atoms are then clustered using a single-linkage clustering algorithm with a distance threshold of 8 Å. Each cluster is used to identify the possible superpositions to the donor atoms of a template MBS, with an approach analogous to template-based docking. All of the possible permutations of donor atoms are evaluated, and only those featuring an RMSD lower than 0.5 Å between the donor atoms of the template and of the AlphaFold model are retained to be checked for steric clashes between the cofactor and the protein atoms. After rejecting all of the permutations with poor RMSDs or steric clashes, the permutation with the lowest RMSD is retained for the current cluster of potential donor atoms. Twelve different template MBSs were analyzed in the work, six for iron–sulfur clusters and six for different zinc(II) sites, containing a single ion with three or four donor atoms. In practice, by looking at whether the sidechain atoms in each AlphaFold model were pre-organized to allow one of the twelve template MBSs to be docked to the protein with a low RMSD and no clashes, the authors predicted as many as 13,139-binding sites in 7490 unique proteins with no known structural homologs [93]. The concept is similar to the template-based docking approach depicted in Figure 2, except that the apo-structure is taken from AlphaFold predictions rather than being solved experimentally. Intriguingly, the above repertoire might be even larger if one takes into account the fact that proteins can populate different conformational states, while AlphaFold predicts only a single state [94].

The bindEmbed21 approach combines homology-based inference with ML to predict whether a protein residue binds to a metal ion, a nucleic acid, or a small molecule [95]. The ML component used protein embeddings as inputs to a two-layer convolutional neural network (CNN). Protein embeddings consist of fixed-length vector representations for each residue in a sequence, based on the distribution of sequences in an unlabeled set (i.e., a sequence database of proteins without experimental characterization whatsoever). In practice, this type of representation embeds each protein sequence in a vector space and allows the CNN to learn the constraints of a protein sequence [96]. The advantage of this approach is that it does not require knowledge of protein structures, which is scarcer than the knowledge of protein sequences; nor expert-selected features, which may require prior information on the chemicophysical properties which are relevant for the problem of interest; nor evolutionary information derived from multiple sequence alignments (MSAs), which are computationally cumbersome. The overall performance of bindEmbed21 was close to that of specialized zinc-binding prediction methods, including the Zincbindpredict tool described in Section 3.2.

Mebipred is a tool to predict whether a protein is an MP based on sequence information alone [97]. It is relevant for this review because it was trained using information derived from 3D structures. This tool exploits a feed-forward multi-layer perceptron, a specific type of NN. The training data of mebipred were built on the MP structures available from the PDB, clustered at 70% sequence identity. For each representative structure, the input features were the amino acid composition, the physicochemical properties of the amino acids in the sequence, and the frequency of occurrence of pentameric residue sequences within 5 Å of the metal ion. When analyzing a new query sequence, the latter is decomposed into pentamers with a sliding window of one position, and the structure-derived feature is calculated as the sum of the occurrences of the pentamers in the PDB dictionary. In practice, mebipred looks in the sequence for pentamers that were detected previously in the entire PDB database as being within 5 Å of the metal in a MBS of known structure. Based on the pentamers detected in the query, the chemical identity of the bound metal can be predicted as well, based on the metal content of the 3D structures where these pentamers occurred. Mebipred can identify MPs with an 80% accuracy and can define the chemical identities of 11 different metals, for which a sufficient number of PDB structures were available.

In a very recent application, our own research team developed a DL classifier that can discriminate physiological and adventitious zinc-binding sites in the 3D structures of MPs with an accuracy of about 90% [98]. In order to develop the tool, we trained a recurrent neural network (RNN) using a dataset of 1944 physiological and 3352 adventitious zinc-binding sites extracted from MetalPDB and manually annotated. In order to compensate for the imbalance with respect to adventitious sites, the weight of the physiological sites in the cost function of the RNN was scaled up by 1.7. In addition to zinc-binding sites, the same DL classifier (i.e., without further training) could discriminate non-heme mononuclear iron sites with an accuracy close to 80%. This indicates that the rules learnt on zinc sites have general relevance, at least for simple transition metal ions. By systematically evaluating the importance of the various features input to the DL classifier, it appeared that MBSs involving 20 protein residues or more (defined according to the protocol of Figure 1) are quite likely to be physiological. The same holds for sites with four metal ligands or more provided by the protein chain. Furthermore, it was observed that metal ligands in physiological MBSs tend to be buried, as judged from their relatively low solvent accessibility.

## 7. Conclusions

The extensive information available on MP structures has enabled the development of a multitude of applications for a deeper understanding of the biochemistry of MPs (Table 1). The tools for the prediction of the occurrence of MBSs in apo-structures and in structural models lacking their metal cofactor allow researchers to obtain a complete view of the occurrence of MPs in different organisms. The systematic structural comparison of these MBSs then results in the identification of distant evolutionary relationships, which would go unnoticed with other methods, or highlights cases of evolutionary convergence. A blooming sector is the application of ML and DL methods to MPs, which is providing unprecedented insight into the structure–function relationship in these systems. This whole plethora of computational advances is supported by public databases derived from the PDB and integrating specialized functional information together with systematic analyses for selected aspects of the biochemistry of MPs. We are fully confident that this growth trend will be reinforced in the coming years, leading to an unprecedented level of comprehension of the role of essential metal ions in living organisms.

Table 1 summarizes the resources and applications mentioned in this contribution, in the order in which they were described.

## Figures and Tables

**Table 1:** Summary of all of the resources mentioned in this article. The resources are listed in the same order as they are discussed in the corresponding sections. Only links active as of 15 June 2022 are included.

Tool Name and Link	Implemented Approach	Reference
**Template-Based Methods**		
	Identification of cavities with high hydrophobicity contrast	[33]
CHED	Identification of suitable arrangement(s) of triads of the CHED residues based on the distances between candidate donor atom	[36]
IonCom https://zhanggroup.org/IonCom/ (accessed on 5 July 2022)	Integration of four structure-based predictors and a novel sequence-based predictor	[38]
MIB http://bioinfo.cmu.edu.tw/MIB/ (accessed on 5 July 2022)	Docking MBS templates with the fragment transformation method	[41]
ZINCCLUSTER http://www.metalactive.in/ (accessed on 5 July 2022)	Detection of known structural patterns	[43]
Predictive algorithm in the GaudiMM modeling suite	Identification of accessible cavities whose center of mass is within 3.5 Å from the β-carbon atoms of three or more CHED residues	[45]
BioMetAll https://github.com/insilichem/biometall (accessed on 5 July 2022)	Identification of cavities followed by their validation against pre-defined geometric patterns of the protein backbone	[46]
N.A.	Docking MBS templates with geometric hashing against an ensemble of 11 structural conformations for the query protein, generated with coarse-grained molecular mechanics	[48]
**Random forest methods**		
Zincbindpredict https://zincbind.net/predict (accessed on 5 July 2022)	Application of a portfolio of predictive models, each optimized to detect a specific type of zinc-binding site. Each type corresponds to a different zinc-binding patterns.	[49]
	Prediction of positions where metal ligands can be introduced, based on protein backbone coordinates, to design artificial MPs	[50]
**Structural comparison of metal sites**		
MetalS^2^ http://metalweb.cerm.unifi.it/tools/metals2/ (accessed on 5 July 2022)	Pairwise metal-centered superposition of MBSs based on a combination of sequence and structural similarity	[53]
MetalS^3^ http://metalweb.cerm.unifi.it/tools/metals3/ (accessed on 5 July 2022)	A web server using an optimized version of MetalS^2^ to search the MetalPDB database for MBSs structurally similar to the query	[56]
mFASD http://staff.ustc.edu.cn/~liangzhi/mfasd/ (accessed on 5 July 2022)	A structure-based algorithm to predict which metal populates a MBS based on systematic comparison against a template library	[58]
MeCOM https://mecom.ddtmlab.org (accessed on 5 July 2022)	Pairwise superposition of MBSs based on a combination of site features and the position of the Cα atoms	[59]
TopMatch + Sahle https://topmatch.services.came.sbg.ac.at (accessed on 5 July 2022)	Scoring of pairwise structural superpositions computed by the TopMatch tool, which ignores metal ions, with the sahle function to detect alignments having a good overlap of the MBSs	[62]
**Metalloprotein databases**		
MetalPDB https://metalpdb.cerm.unifi.it/ (accessed on 5 July 2022)	MetalPDB collects structural information on all the MBSs present in the Protein Data Bank	[8]
BioLiP https://zhanggroup.org/BioLiP (accessed on 5 July 2022)	A database collecting structures of protein adducts, including metal-protein complexes	[67]
ZincBind https://zincbind.net (accessed on 5 July 2022)	A database specialized on zinc-binding sites built on biological assemblies	[68]
PyDISH https://pydish.bio.info.hiroshima-cu.ac.jp (accessed on 5 July 2022)	PyDISH is specialized on the analysis of heme-binding sites in PDB structures	[69]
VirusMED https://virusmed.biocloud.top (accessed on 5 July 2022)	A database of epitopes, drug binding site and metal binding sites in viral proteins of known 3D structure	[71]
InterMetalDB https://intermetaldb.biotech.uni.wroc.pl (accessed on 5 July 2022)	A database of MBSs occurring at macromolecular interfaces, built on biological assemblies	[30]
MetLigDB http://silver.sejong.ac.kr/MetLigDB (accessed on 4 July 2022)	MetLigDB focuses on the structural and chemical properties of small molecules that bind directly to the metal ion(s) in MP structures	[75]
MeLAD https://melad.ddtmlab.org (accessed on 5 July 2022)	A database derived from the 3D structures of all metalloenzyme-ligand adducts, which integrates detailed analyses of metal-binding pharmacophores, metalloenzyme structural similarity and ligand chemical similarity	[76]
**AI methods applied to metalloproteins**		
https://github.com/psipred/protein-vae (accessed on 5 July 2022)	Use of conditional variational autoencoders for the automated design of artificial metalloproteins	[80]
https://bitbucket.org/mkoohim/multichannel-cnn (accessed on 5 July 2022)	Identification of disease-related mutations through a multichannel convolutional neural network (MCCNN)	[81]
DeepCys https://deepcys.herokuapp.com/ (accessed on 5 July 2022)	Discrimination of four cysteine different roles, i.e., metal-binding, disulphide formation, sulphenylation and thioether	[82]
MAHOMES https://github.com/SluskyLab/MAHOMES (accessed on 5 July 2022)	Discrimination of enzymatic and non-enzymatic metals in MPs	[84]
AlphaFill https://alphafill.eu/ (accessed on 5 July 2022)	A database derived from AlphaFold predictions of apo-proteins where holo-structures of MPs have been reconstructed	[89]
bindEmbed21 https://github.com/Rostlab/bindPredict (accessed on 5 July 2022)	bindEmbed21 uses a combination of homology-based inference and a convolutional neural network to predict whether a protein residue binds to a metal ion, a nucleic acid, or a small molecule	[95]
*mebipred* https://services.bromberglab.org/mebipred (accessed on 5 July 2022)	Sequence-based prediction of MPs using a NN trained with information derived from 3D structures	[97]
https://github.com/cerm-cirmmp/MBSDL (accessed on 5 July 2022)	Discrimination of physiological and adventitious zinc-binding sites in MPs using a recurrent neural network (RNN)	[98]

## Data Availability

No applicable.
